# Protective and Risk Factors in Exercise Addiction: A Series of Moderated Mediation Analyses

**DOI:** 10.3390/ijerph18189706

**Published:** 2021-09-15

**Authors:** Alessio Gori, Eleonora Topino, Mark D. Griffiths

**Affiliations:** 1 Department of Health Sciences, University of Florence, Via di San Salvi 12, Pad. 26, 50135 Florence, Italy; 2 Integrated Psychodynamic Psychotherapy Institute (IPPI), Via Ricasoli 32, 50122 Florence, Italy; 3 Department of Human Sciences, LUMSA University of Rome, Via della Traspontina 21, 00193 Rome, Italy; eleonora.topino@gmail.com; 4 Psychology Department, Nottingham Trent University, 50 Shakespeare Street, Nottingham NG1 4FQ, UK; mark.griffiths@ntu.ac.uk

**Keywords:** exercise addiction, problematic exercise, behavioral addiction, disordered eating, eating disorders, body perception

## Abstract

For a minority of individuals, exercise may become excessive and lead to an addictive behaviour. To better understand the processes by which exercise could become an addiction, the present study examined the risk and protective factors of exercise addiction among regular exercisers, by investigating the role of drive for thinness, bulimia, body dissatisfaction, body image concerns, and self-esteem. A sample of 319 Italian regular exercisers (M_age_ = 30.78 years, SD = 11.98) completed the Italian versions of the Exercise Addiction Inventory, Eating Disorder Inventory-3 Referral Form, Body Image Concern Inventory, and Rosenberg Self-Esteem Scale. Data were analyzed by implementing a series of moderated mediations. Drive for thinness, bulimia, and body dissatisfaction were positively associated with exercise addiction. An indirect path was found in each of these relationships, which included the mediation of body image concerns, as well as a significant moderation of self-esteem in the associations between drive for thinness, bulimia, body dissatisfaction, and the mediator. High self-esteem appeared to be a protective factor. The higher the level of self-esteem, the less indirect the effects of thinness drive, bulimia, body dissatisfaction, and body image concerns were on exercise addiction. Such findings contribute to a better understanding concerning the risk and protective factors of excessive exercise, and may have important practical implications in structuring interventions to reduce risk of developing exercise addiction, as well as orienting future research.

## 1. Introduction

In the fifth edition of the *Diagnostic and Statistical Manual of Mental Disorders*, only Gambling Disorder has officially been recognised as a behavioural addiction (DSM-5) [1]. Nonetheless, it is becoming increasingly evident that some individuals can become addicted to different behaviours that do not involve the ingestion of a psychoactive substance, such as problematic internet use, gaming disorder, compulsive sexual behaviour disorder, compulsive buying, exercise addiction, and work addiction, among others [2]. Among these, exercise addiction has been defined as “*a morbid pattern of behavior in which the habitually exercising individual loses control over his or her exercise habits and acts compulsively, exhibits dependence, and experiences negative consequences to health as well as in his or her social and professional life*” (p. 303) [3]. This definition is also further deepened with the integration of the six components of behavioural addictions [4], i.e., salience, mood modification, tolerance, withdrawal, conflict, and relapse, which have been demonstrated to have a well-founded application to exercise addiction [5,6,7]. Therefore, this condition describes individuals who perform a harmful level of physical activity [8,9] to the point of developing dependence [5,6,9], losing control [10] and perpetuating problematic behaviour, despite the difficulties and compromises associated with it [11,12], and also significantly compromises the personal functioning in different spheres of life (see Berczik et al., [13] for a review). In other words, when the exercise becomes excessive, a minority of individuals may develop adverse health consequences, both physical and psychological [14]. Physical damage is manifested predominantly through long-term risks such as musculoskeletal pain, stress fractures, and repeated injuries [15], while psychological damage is typically expressed with some degree of negative emotional and physiological symptoms [16], as well as through changes in mood, such as the feeling of depression, anxiety, guilt, irritability, and lacking energy, when the individual cannot exercise [17]. Since the motivation to exercise seems to be a key factor in identifying individuals at risk of developing exercise addiction [6,18,19], the present study explored the risk and protective factors of exercise addiction among regular exercisers by investigating the roles of drive for thinness, bulimia, body dissatisfaction, body image concerns, and self-esteem. 

The variables associated with eating disorders, as well as body dysmorphism, may plausibly be risk factors for exercise addiction, since the tendency towards a morbid modality of exercise may be favoured by the individual’s goals and expectations associated with those behaviours [20], including those concerning the improvement of one’s body [21,22]. Concerning eating disorders, although exercise addiction is sometimes considered as a primary condition, other research has also investigated it as a secondary condition, showing that these behaviours have similar symptoms and consequences [18,23,24]. In this regard, a recent estimate reported greater risk of exercise addiction as a comorbidity to an eating disorder than among individuals that do not report this disease [23], since exercise activity may represent a way to control and favour specific physical characteristics [25]. 

Among the aspects associated with eating disorders, drive for thinness, bulimia, and body dissatisfaction have been considered key elements [26,27]. Previous research has shown significant associations between drive for thinness with pathological exercise [28]. Indeed, drive for thinness indicates the tendency to desire (in an extreme way) to be thin and a fear of gaining weight, as well as attention and concerns about diet [26,27], and has consistently been found to be a significant predictor of exercise addiction in physical activities where weight loss is one of the primary goals [29,30]. Similarly, research has also focused on the effects of symptoms related to bulimia, defined by the presence of episodes in which large quantities of food are consumed in an uncontrolled way (bingeing), with consequent emotional agitation and psychological malaise [26,27]. Previous evidence has highlighted an association between bulimia, exercise commitment, and fixation, indicating a certain degree of negative emotionality associated with missing an exercise session, and the tendency to use physical activity to compensate for overeating [31]. In relation to body dissatisfaction, the literature has shown an association with risk behaviour for addiction to exercise [32], even among populations with no referred eating disorders (e.g., [23,33]). Body dissatisfaction may be defined as a state of displeasure concerning the overall shape and size of one’s own body in general, as well as of those regions that are of greatest concern to those with eating disorders (e.g., buttocks, stomach, thighs, hips) [26,27]. These aspects may favour morbid exercise modalities through both negative reinforcement (e.g., to avoid the guilt of not having done enough to compensate for the caloric intake), and positive reinforcement (e.g., to obtain a more toned or thin body) [34,35].

Moreover, body image perception reflects the evaluation of one’s body as positive or negative and may be a significant motivator for exercise [36]. It is a complex and multidimensional construct indicating thoughts, perceptions, and feelings of one’s own body [37]. Individuals who report excessive concerns over their physical appearance and weight are most likely to develop exercise addiction [38]. Body image concerns comprise a preoccupation with, checking for and/or trying to camouflage a perceived physical defect [39], and have been found to be a frequent symptom among those with eating disorders [40], as well as being associated with more serious cases of exercise addiction [41]. 

On the other hand, not all individuals with disordered eating patterns engage in physical exercise compulsively [42], and this in part may be due to the existence of other protective factors that make the perception of their own body image less problematic. In this regard, previous evidence identified significant differences in the levels of self-esteem between those with exercise addiction and the control group, with higher levels in the latter [43]. Self-esteem may be defined as the overall value a person places on the self [44]. It may have an influence on body image-related preoccupation [45,46] and has shown significant associations with a more positive self-concept and better mental health [45]. Previous studies have consistently shown a strong association between self-esteem and body image among individuals with eating disorders to a greater extent than among patients with other psychiatric disorders [47].

### Objectives and Hypotheses 

**Figure 1:**
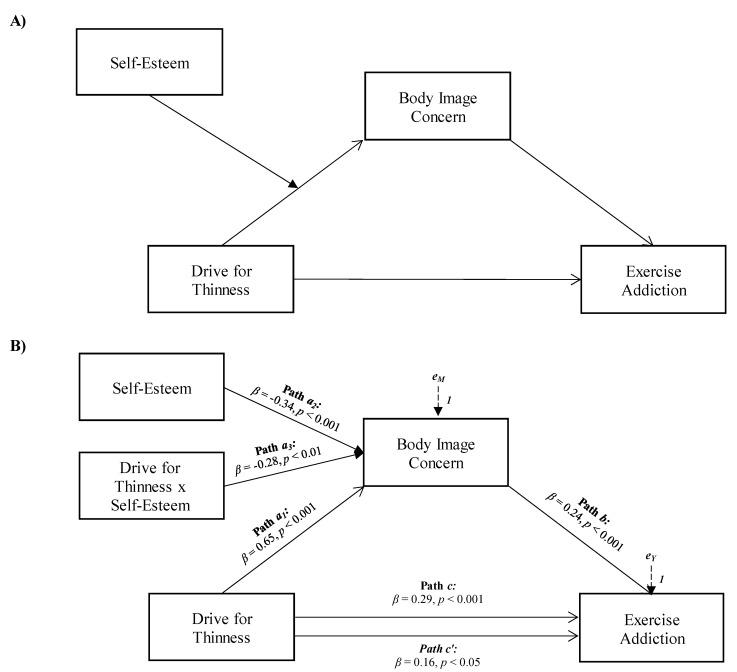
Statistical (**A**) and conceptual (**B**) forms of Model 1: A moderated mediation involving drive for thinness, body image concern, and self-esteem toward exercise addiction.

Taking these aforementioned findings into account, in the present study, three moderated mediation models were elaborated to delve deeper into the existing knowledge concerning exercise addiction, by investigating its relationships with the factors that may influence exercise addiction among regular exercisers. More specifically, the first aim was to explore the role of drive for thinness, body image concerns, and self-esteem in contributing to exercise addiction. Based on the presented theoretical framework, it was hypothesized that (see Figure 1A):

**Hypothesis** **1** **(H_1_).** *Drive for thinness will be associated with exercise addiction*;

**Hypothesis** **2** **(H_2_).** *Body image concerns will positively mediate the association between drive for thinness and exercise addiction*;

**Hypothesis** **3** **(H_3_).** *Self-esteem will moderate the relationships between drive for thinness and body image concerns*.

**Figure 2:**
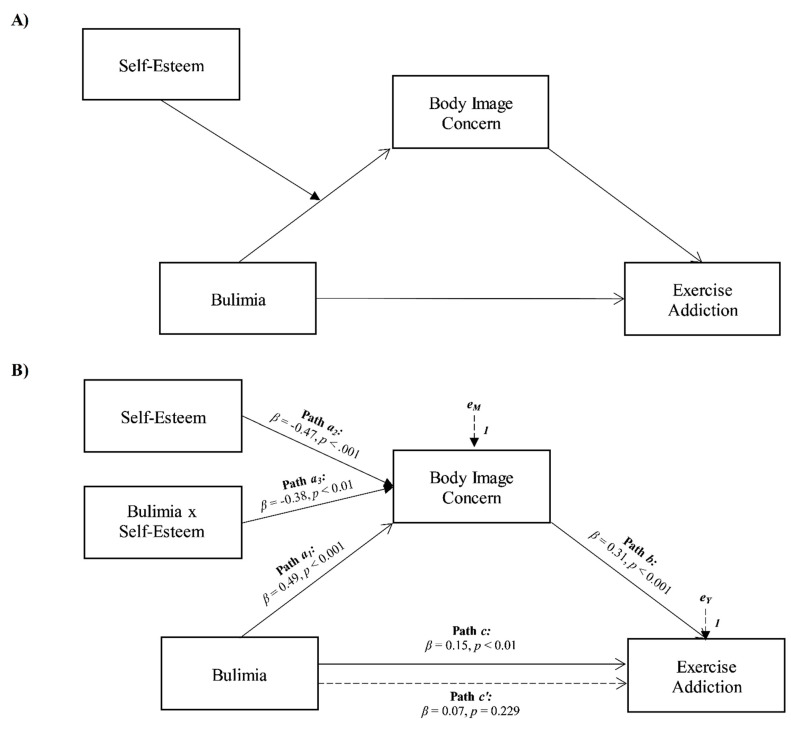
Statistical (**A**) and conceptual (**B**) forms of Model 2: a moderated mediation involving bulimia, body image concern, and self-esteem toward exercise addiction.

The second aim was to explore the role of bulimia, body image concerns, and self-esteem in contributing to exercise addiction. Based on the presented theoretical framework, it was hypothesized that (see Figure 2A):

**Hypothesis** **4** **(H_4_).** *Bulimia will be associated with exercise addiction*;

**Hypothesis** **5** **(H_5_).** *Body image concerns will positively mediate the association between bulimia and exercise addiction*;

**Hypothesis** **6** **(H_6_).** *Self-esteem will moderate the relationships between bulimia and body image concerns*.

**Figure 3:**
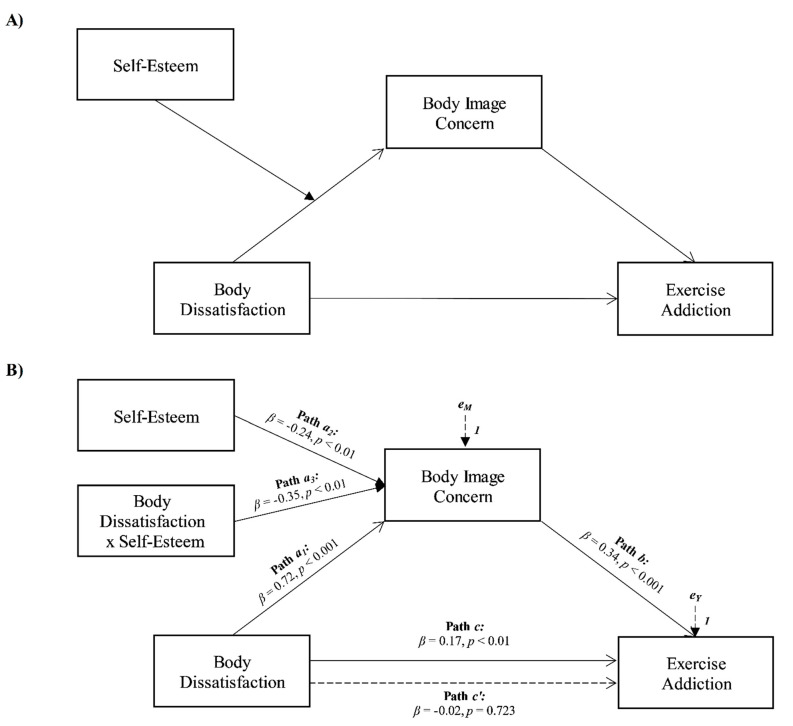
Statistical (**A**) and conceptual (**B**) forms of Model 3: a moderated mediation involving body dissatisfaction, body image concern, and self-esteem toward exercise addiction.

The third aim was to explore the role of body dissatisfaction, body image concerns, and self-esteem in contributing to exercise addiction. Based on the presented theoretical framework, it was hypothesized that (see Figure 3A):

**Hypothesis** **7** **(H_7_).** *Body dissatisfaction will be associated with exercise addiction*;

**Hypothesis** **8** **(H_8_).** *Body image concerns will positively mediate the association between body dissatisfaction and exercise addiction*;

**Hypothesis** **9** **(H_9_).** *Self-esteem will moderate the relationships between body dissatisfaction and body image concerns*.

## 2. Materials and Methods

### 2.1. Participants, Procedure, and Ethics

The sample comprised 319 participants (71% females and 29% males) who engaged in regular exercise (i.e., at least three times per week for a minimum of 30 minutes each session). The mean age was 30.78 years (*SD* = 11.98; age range: 18–75 years). Most of the participants were single (69%), had a high school diploma (45%), and were students (33%) (Table 1). Using snowball sampling, they were recruited online by sending out a link to the online survey hosted on the *Google Forms* platform. There was no payment for participating in the study and all participants were volunteers. Before starting the survey, all participants were informed about the general aim of the research and that their answers would be used for research purposes in an aggregated way. Privacy and anonymity were ensured. They were also told that they could leave the study at any time. Furthermore, they provided informed consent electronically. The study was approved by the Ethical Committee of the Integrated Psychodynamic Psychotherapy Institute (IPPI).

### 2.2. Measures

*Exercise Addiction Inventory* (EAI; Griffiths, Szabo, and Terry [48]; Terry, Szabo, and Griffiths [9]; Italian version: Gori, Topino, and Griffiths [49]). The six-item EAI was used to assess the risk of exercise addiction, with items based on the components model of behavioural addiction [5]. Items (e.g., “*If I have to miss an exercise session, I feel moody and irritable*”) rated on a five-point response option ranging from 1 (“*strongly disagree*”) to 5 (“*strongly agree*”). In the present study, the EAI exhibited good validity (*χ^2^* = 20.50, *p* < 0.05, NNFI = 0.94, CFI = 0.96, RMSEA = 0.06, SRMR = 0.04) and reliability (α = 0.71).

*Eating Disorder Inventory-3-Referral Form* (EDI-3-RF; Garner [50]; Italian version: Giannini and Conti [27]). The 25-item EDI-3-RF was used to assess disordered eating symptomatology. Items are rated on a six-point response option (from A = “*always*”; to F = “*never*”, with a 0–4 scoring system, with two responses in the non-symptomatic direction both scoring 0) [27], and comprise three subdimensions: drive for thinness (DT; e.g., “*I think about dieting*”), bulimia (B; e.g., “*I eat moderately in front of others and stuff myself when they’re gone*”), and body dissatisfaction (BD; e.g., “*I think that my stomach is too big*”). A second section in the scale provides criteria for referral to a specialist based on: (i) the Body Mass Index together with gender and age (BMI); (ii) the BMI in relationship to the scores on the DT and B subscales; and (iii) the presence behavioural symptoms related to extreme weight control modes (e.g., self-induced vomiting). The endorsement of at least one of the three referral criteria indicates the risk of an eating disorder. In the present study, EDI-3-RF exhibited acceptable validity (*χ^2^* = 555.39, *p* < 0.001, NNFI = 0.90, CFI = 0.92, RMSEA = 0.07, SRMR = 0.08) and very good to excellent levels of reliability for its subscales (DT α = 0.91; B α = 0.88; BD α = 0.85).

*Body Image Concern Inventory* (BICI) (Littleton, Axsom, and Pury [39]; Italian version: Luca et al., [51]). The 19-item BICI was used to assess dysmorphic body image concerns. Items (e.g., “*I feel there are certain aspects of my appearance I would like to change*”) are rated on a five-point response option ranging from 1 (“*never*”) to 5 (“*always*”). In the present study, the BICI exhibited acceptable validity (*χ^2^* = 407.72, *p* < 0.001, NNFI = 0.90, CFI = 0.93, RMSEA = 0.09, SRMR = 0.06) and an excellent level of reliability (α = 0.94).

*Rosenberg Self-Esteem Scale* (RSES: Rosenberg [44]; Italian version: Prezza, Trombaccia, and Armento [52]). The 10-item RSES was used to assess global self-esteem. Items (e.g., “*On the whole, I am satisfied with myself*”) are rated on a four-point option (ranging from 0 = “*strongly disagree*”, to 3 = “*strongly agree*”). In the present study, the RSES exhibited excellent validity (*χ^2^* = 60.544, *p* < 0.001, NNFI = 0.96, CFI = 0.98, RMSEA = 0.08, SRMR = 0.04) and reliability (α = 0.91).

### 2.3. Data Analysis

All the statistical analyses were conducted using SPSS 21.0 (IBM, Armonk, NY, USA) for Windows. Descriptive statistics were carried out. Then, Pearson’s *r* correlation analyses were performed to investigate the associations between the variables. An independent samples *t*-test was carried out to explore the differences in the scores of exercise addiction between participants who reported at least one referral criterion of the EDI-3-RF (and may therefore be at risk of an eating disorder) and those who did not. To assess the hypothesized roles of drive for thinness, bulimia, body dissatisfaction, body image concerns, and self-esteem on exercise addiction, several moderated mediations were tested using Model 7 in the macro-program PROCESS 3.4 [53]. A 95% confidence interval (CI) was calculated for each regression coefficient included in the model. Finally, the statistical stability of the models was estimated by testing the conditional indirect effect following the Wayne et al. method [54], and by performing the bootstrapping procedure for each of the 5000 bootstrapped samples with a 95% confidence interval. If the interval (from lower limit confidence interval [Boot LLCI] to upper limit confidence interval [Boot ULCI]) does not include zero, the indirect effect is considered to be statistically significant. A two-tailed value of *p* < 0.05 was the level of statistical significance set in the present study.

## 3. Results

Descriptive statistics for the study sample are presented in Table 1. Furthermore, means and standard deviations of the scales used were calculated: Exercise addiction Inventory (EAI), *M* = 17.27, *SD* = 4.56; Drive for Thinness (EDI-3-RF), *M* = 12.77, *SD* = 10.26; Bulimia (EDI-3-RF), *M* = 6.54, *SD* = 7.72; Body Dissatisfaction (EDI-3-RF), *M* = 11.86, *SD* = 8.25; Body Image Concern Inventory (BICI), *M* = 46.57, *SD* = 16.88; and Rosenberg Self-Esteem Scale (RSES), *M* = 20.76, *SD* = 6.90. 

Pearson’s *r* correlation analyses (see Table 2) showed significant and positive associations between exercise addiction scores and those of drive for thinness (*r* = 0.242, *p* < 0.01), bulimia (*r* = 0.150, *p* < 0.01), body dissatisfaction (*r* = 0.172, *p* < 0.01), and body image concerns (*r* = 0.328, *p* < 0.01). The latter, in turn, was also significantly and positively associated with drive for thinness (*r* = 0.656, *p* < 0.01), bulimia (*r* = 0.269, *p* < 0.01) and body dissatisfaction (*r* = 0.573, *p* < 0.01). Self-esteem significantly and negatively correlated with all the other variables: exercise addiction (*r* = −0.203, *p* < 0.01), drive for thinness (*r* = −0.396, *p* < 0.01), bulimia (*r* = −0.230, *p* < 0.01), body dissatisfaction (*r* = −0.565, *p* < 0.01), and body image concerns (*r* =−0.631, *p* < 0.01). 

The independent samples *t*-test showed significantly higher scores for exercise addiction among participants who reported at least one referral criterion (*N* = 116; *M* = 16.55, *SD* = 4.43) than those who did not (*N* = 203; *M* = 17.68, *SD* = 4.59): *t*(317) = 2.157, *p* < 0.05. 

**Figure 4:**
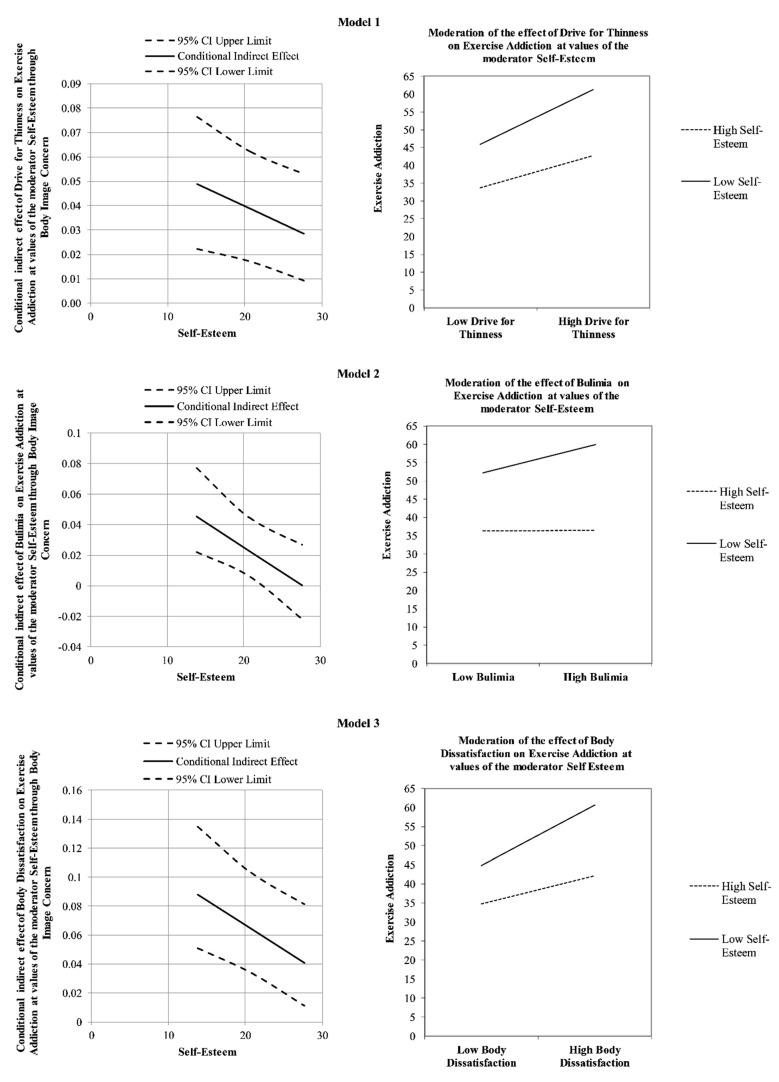
Graphic representation of the moderation effect for each Model.

The results of a first moderated mediation analysis confirmed that body image concerns partially mediated the relationship between drive for thinness and exercise addiction, and the association between drive for thinness and body image concerns was moderated by self-esteem (see Figure 1). More specifically, drive for thinness was positively and significantly associated with exercise addiction (path *c* in Figure 1B; *β* = 0.29, *p* < 0.001, LLCI = 0.0826–ULCI = 0.1765) and body image concerns, the mediator variable (path *a* in Figure 1B; *β* = 0.65, *p* < 0.001, LLCI = 0.7012–ULCI = 1.4406). Furthermore, body image concerns were significantly associated with exercise addiction (path *b* in Figure 1B; *β* = 0.24, *p* < 0.001, LLCI = 0.0308–ULCI = 0.0986), and when included in the model, it partially mediated the effect of drive for thinness on exercise addiction, reducing the direct effect, but which remained significant (path *c’* in Figure 1B; *β* = 0.16, *p* < 0.05, LLCI = 0.0136–ULCI = 0.1252): *R^2^* = 0.124, *F*(2, 316) = 22.379, *p* < 0.001. Self-esteem was found to interact negatively and significantly with drive for thinness towards body image concerns (path *a_3_* in Figure 1B; *β* = −0.28, *p* < 0.01, LLCI = −0.0400–ULCI = −0.0056), and the index of moderated mediation was found to be significant (Index = −0.0015, Boot LLCI = −0.0031–Boot ULCI = −0.0002). Furthermore, the interaction was further investigated by testing the conditional effects of drive for thinness at three levels of self-esteem (i.e., −1SD, mean, and +1SD). The association between drive for thinness and body image concerns was slightly stronger at low levels of self-esteem (estimate = 0.755(0.09), *p* < 0.001, LLCI = 0.5823–ULCI = 0.9270), than at average levels (estimate = 0.597(0.07), *p* < 0.001, LLCI = 0.4597–ULCI = 0.7344) or high levels (estimate = 0.439(0.10), *p* < 0.001, LLCI = 0.2489–ULCI = 0.6299). Therefore, when participants reported higher levels of self-esteem, the positive indirect effect of drive for thinness on exercise addiction via body image concerns weakened (see Model 1 in Figure 4). Finally, the bootstrap analysis confirmed the statistical relevance and robustness of the moderation effect: Boot LLCI = −0.0418–Boot ULCI = −0.0043. 

Moreover, the results of a second moderated mediation analysis confirmed that body image concerns totally mediated the relationship between bulimia and exercise addiction, and the association between bulimia and body image concerns was moderated by self-esteem (see Figure 2).

More specifically, bulimia was positively and significantly associated with exercise addiction (path *c* in Figure 2B; *β* = 0.15, *p* < 0.01, LLCI = 0.0239–ULCI = 0.1530) and body image concerns, the mediator variable (path *a* in Figure 2B; *β* = 0.49, *p* < 0.001, LLCI = 0.5583–ULCI = 1.6037). Furthermore, body image concerns were significantly associated with exercise addiction (path *b* in Figure 2B; *β* = 0.31, *p* < 0.001, LLCI = 0.0545–ULCI = 0.1130), and when included in the model, it totally mediated the effect of bulimia on exercise addiction, since the direct effect became non-significant (path *c’* in Figure 2B; *β* = 0.07, *p* = 0.229, LLCI = −0.0248–ULCI = 0.1032; *R^2^* = 0.112, *F*(2, 316) = 19.837, *p* < 0.001). Self-esteem was found to interact negatively and significantly with bulimia towards body image concerns (path *a_3_* in Figure 2B; *β* = −0.38, *p* < 0.01, LLCI = −0.0629–ULCI = −0.0150), and the index of moderated mediation was found to be significant (Index = −0.0033, Boot LLCI = −0.0060–Boot ULCI = −0.0010). Furthermore, the interaction was further investigated by testing the conditional effects of bulimia at three levels of self-esteem (i.e., −1SD, mean, and +1SD). The association between bulimia and body image concerns was slightly stronger at low levels of self-esteem (estimate = 0.542[0.12], *p* < 0.001, LLCI = 0.2974–ULCI = 0.7861) than at average levels (estimate = 0.273[0.10], *p* < 0.01, LLCI = 0.0851–ULCI = 0.4609), and became non-significant at high levels (estimate = 0.004[0.13], *p* = 0.974, LLCI = −0.2515–ULCI = 0.2600). Therefore, when participants reported higher levels of self-esteem, the positive indirect effect of bulimia on exercise addiction via body image concerns weakened to become non-significant (see Model 2 in Figure 4). Finally, the bootstrap analysis confirmed the statistical relevance and robustness of the moderation effect: Boot LLCI = −0.0676 Boot ULCI = −0.0129.

Finally, the results of a third moderated mediation analysis confirmed that body image concerns totally mediated the relationship between body dissatisfaction and exercise addiction, and the association between body dissatisfaction and body image concerns was moderated by self-esteem (see Figure 3).

More specifically, body dissatisfaction was positively and significantly associated with exercise addiction (path *c* in Figure 3B; *β* = 0.17, *p* < 0.01, LLCI = 0.0350–ULCI = 0.1553) and body image concerns, the mediator variable (path *a* in Figure 2B; *β* = 0.72, *p* < 0.001, LLCI = 0.9775–ULCI = 1.9705). Furthermore, body image concern was significantly related to exercise addiction (path *b* in Figure 3B; *β* = 0.34, *p* < 0.001, LLCI = 0.0577–ULCI = 0.1266), and when included in the model, it totally mediated the effect of body dissatisfaction on exercise addiction, since the direct effect became non-significant (path *c’* in Figure 3B; *β* = −0.02, *p* = 0.723, LLCI = −0.0832–ULCI = 0.0578; *R^2^* = 0.109, *F*(2, 316) = 19.094, *p* < 0.001). Self-esteem was found to interact negatively and significantly with body dissatisfaction towards body image concerns (path *a_3_* in Figure 3B; *β* = −0.35, *p* < 0.001, LLCI = −0.0605–ULCI = −0.0141), and the index of moderated mediation was found to be significant (Index = −0.0034, Boot LLCI = −0.0062–Boot ULCI = −0.0012). Furthermore, the interaction was further investigated by testing the conditional effects of body dissatisfaction at three levels of self-esteem (i.e., −1SD, mean, and +1SD). The association between body dissatisfaction and body image concerns was slightly stronger at low levels of self-esteem (estimate = 0.957[0.11], *p* < 0.001, LLCI = 0.7285–ULCI = 1.1850), than at average levels (estimate = 0.699[0.09], *p* < 0.001, LLCI = 0.5192–ULCI = 0.8788) and high levels (estimate = 0.441[0.13], *p* < 0.001, LLCI = 0.1885–ULCI = 0.6030). Therefore, when participants reported higher levels of self-esteem, the positive indirect effect of body dissatisfaction on exercise addiction via body image concerns weakened (see Model 3 in Figure 4). Finally, the bootstrap analysis confirmed the statistical relevance and robustness of the moderation effect: Boot LLCI = −0.0641; Boot ULCI = −0.0138. The main indices concerning the effects of model are summarized in Table 3.

## 4. Discussion

Similar to other behavioural addictions, several studies have shown that physical exercise may result in dysfunctional behaviour, leading to negative consequences and damage on physical, psychological and social levels [17]. Although exercise remains a behaviour to be promoted to improve health, extreme exercise needs to be investigated in relation to the symptomatology of addiction [24]. Within this framework, the present study explored the risk and protective factors of exercise addiction among regular exercisers, by examining the role of drive for thinness, bulimia, body dissatisfaction, body image concerns, and self-esteem.

### 4.1. Differences in the Levels of Exercise Addiction Based on the Risk of Eating Disorder 

The results indicated that just over half of the regular exercisers reported at last one referral criterion on the EDI-3-RF, and that they scored significantly higher for the risk of exercise addiction than those who do not present elements of being at risk of eating disorders. These data support that, even if exercise addiction exists as a primary condition associated with a motivation to reduce psychological distress [55], some elements related to eating disorder may be risk factors for exercise addiction and for its severity [56,57]. A recent meta-analysis highlighted a three times higher risk of developing exercise addiction among individuals with scores above the cut-off for eating disorders than among those without an indicated eating disorder [23], with an associated severity of medical conditions related to excessive physical activity (e.g., fractures) [58]. Indeed, unhealthy exercise can be seen as an integral part of these pathologies, functioning as a way to compensate for calorie intake and control the size of one’s own body in a population characterized by extreme aversions of weight gain and obsessions about it [59].

### 4.2. Drive for Thinness as Antecedent of Exercise Addiction

The first aim of the present study was to explore the role of drive for thinness, body image concerns, and self-esteem in contributing to exercise addiction, by performing a moderated mediation analysis. Results showed that all the hypothesized relationships of the first model were confirmed. 

First, drive for thinness was positively associated with exercise addiction (H_1_). These data can be read considering how the weight loss achievable with exercise can be a coveted goal for individuals with high levels of drive for thinness. Therefore, this behaviour–reward association could lead to addiction to physical activity [5,60]. Furthermore, the scientific literature has consistently identified the thin ideal as a predictor of compulsive exercise pattern [61], as well as a reported significant correlation between concerns about weight gain and exercise addiction [29]. The data also demonstrated that the relationship between drive for thinness and exercise addiction was partially mediated by body image concerns (H_2_). This appears consistent with previous studies on patients with anorexia nervosa (of which drive for thinness is a core element) [62], where a high risk of developing exercise addiction has been indicated for individuals with this condition [63], as well as a negative attitude towards their physical dimension and dysfunctional coping strategies [64], of which the morbid exercise aimed at relieving distress could be a manifestation [55]. Finally, self-esteem moderated the effect of drive for thinness on body image concerns (H_3_), so that as self-esteem grows, the effect diminished, albeit still significant. In other words, the data show that the influence of drive for thinness on concerns about one’s physical appearance was found to be attenuated among individuals who had a more positive self-concept. Self-esteem, coherently, has in fact among its components that of body esteem, indicating those aspects of the conception of the body self-related to personal identity [65].

### 4.3. Bulimia as Antecedent of Exercise Addiction

The second aim of the present study was to explore the role of bulimia, body image concerns, and self-esteem in contributing to exercise addiction, by performing a moderated mediation analysis. Results showed that all the hypothesized relationships of the second model have been confirmed. 

First, bulimia was positively associated with exercise addiction (H_4_). This is consistent with previous research identifying significant associations between compulsive exercise and bulimic tendencies [66,67]. The data also demonstrated that the relationship between bulimia and exercise addiction was totally mediated by body image concerns (H_5_). This result could therefore indicate that, within the framework of this symptomatological condition, compulsive exercise would seem primarily related to the component of negative body perception. This is in line with previous studies, which highlights how individuals suffering from bulimia nervosa tend to be concerned about the possibility of unwanted physical aspects that may arise or as a result of their binges in the absence of physical activity [68]. Finally, self-esteem moderated the effect of bulimia on body image concerns (H_6_), so that as self-esteem increases, the effect diminishes, becoming non-significant. In other words, morbid physical activity among individuals with bulimic symptoms can be read as a way to regulate the discomfort deriving from concerns about body appearance [55,67], which lose their salience among individuals with higher levels of self-esteem.

### 4.4. Body Dissatisfaction as Antecedent of Exercise Addiction

The third aim of the present study was to explore the role of body dissatisfaction, body image concerns and self-esteem in contributing to exercise addiction, by performing a moderated mediation analysis. Results showed that all the hypothesized relationships of the third model have been confirmed. 

First, body dissatisfaction was positively associated with exercise addiction (H_7_). This is consistent with the previous research highlighting that the athletic-ideal internalization, dissatisfaction with own body shape and desire to achieve an athletic and muscular physique predicted compulsive exercise patterns [61,67,69]. The data also demonstrated that the relationship between body dissatisfaction and exercise addiction was totally mediated by body image concerns (H_8_). This result could therefore indicate that dissatisfaction with one’s body may determine concerns about one’s physical appearance, that may lead to compulsive forms of exercise, which could therefore be seen as a way to react to the resulting distress [55,70]. Finally, self-esteem moderated the effect of body dissatisfaction on body image concerns (H_9_), so that at higher levels of self-esteem the effect was lower, albeit still significant. Indeed, as supported by scientific literature, self-esteem showed a negative association with concerns on physical appearance [45,46], therefore resulting in a factor which may mitigate the effect of body dissatisfaction.

### 4.5. A Focus on the Mediating Role of Body Image Concerns

The results of the present study showed that the influence of drive for thinness, bulimia, and body dissatisfaction on exercise addiction involve an indirect path, through the effect of body image concerns (which was a total mediator in the associations of bulimia and body dissatisfaction, and a partial mediator in that of drive for thinness). This is consistent with previous research showing that individuals with eating disorders experience higher dysmorphic concerns [40] and higher body image disorders [71]. In turn, lower scores in body appreciation and higher concerns about individual physical appearance have been associated with more severe levels of exercise addiction [41,72]. 

### 4.6. A Focus on the Protective Role of Self-Esteem (the Moderator Variable)

Findings of the present research indicated that self-esteem intervened in the indirect paths of drive for thinness, bulimia, and body dissatisfaction, by reducing the effects on body image concerns, therefore indicating it to be an important protective factor. Furthermore, regarding bulimia, for which the direct effect on exercise addiction scores became non-significant when the mediating effect was considered in the model, at high levels of self-esteem, the indirect path was no longer significant and the effect on exercise addiction was neutralized. This is consistent with previous studies which found a negative relationship between self-esteem and morbid exercise patterns, such that as self-esteem decreases, the risk of exercising addiction increases [33,43]. The findings of the present study enrich previous literature, which highlighted the role of self-esteem in influencing the physical self-concept and, more generally, mental health [65,73,74].

### 4.7. Limitations and Suggestions for Future Research

The present study has some limitations that need to be taken into account when interpreting the findings. First, a modestly sized convenience sample of regular exercisers was recruited utilizing a snowball sampling method. In other words, the sample was self-selecting, unrepresentative, and perhaps only those who were interested and motivated in the topic under investigation completed the survey. Therefore, it would be useful for future research to replicate the study with larger more representative samples and utilizing different recruitment techniques. Furthermore, only self-report measures were used to collect the data, and such data are known to include well-established methods biases, such as social desirability and memory recall. Integration with other kinds of instruments (e.g., actual behavioural measures) in a multimethod approach is recommended for future research to help overcome this issue. Finally, the cross-sectional design of this study requires caution in interpreting causality. Future research would need to implement longitudinal designs in order to determine the specific directions of the relationships examined in the present study.

## 5. Conclusions

The psychophysical benefits of exercise are widely recognized [75]. However, exercise in its most extreme form can also lead to addictive behaviours with potentially harmful consequences for a minority of individuals. The study of the relationship between the associated factors in the present study may contribute to a better understanding of the processes by which exercise could become an addiction for some individuals. Within this framework, the present study focused on various risk factors for exercise addiction, and found relationships relating to drive for thinness, bulimia, body dissatisfaction, body image concerns, as well as an important protective role of self-esteem. Such results may have important practical implications in structuring tailored clinical or preventive interventions, as well as providing insights for future research.

## Figures and Tables

**Table 1:** Demographic characteristics of the sample (*N* = 319).

Characteristics		*M* ± *SD*	*n*	%
*Age* (years)		30.8 ± 11.98		
*Sex*				
	Females		228	71.47
	Males		91	28.53
*Marital Status*				
	Single		220	68.97
	Married		42	13.17
	Cohabiting		44	13.79
	Separated		7	2.19
	Divorced		5	1.57
	Widowed		1	0.31
*Education*				
	Middle School diploma		13	4.08
	High School diploma		145	45.45
	University degree		90	28.21
	Master’s degree		45	14.11
	Post-lauream specialization		26	8.15
*Occupation*				
	Student		104	32.60
	Working student		58	18.18
	Employee		86	26.96
	Freelance		19	5.96
	Entrepreneur		17	5.33
	Trader		8	2.51
	Artisan		5	1.57
	Armed forces		1	0.31
	Unemployed		13	4.08
	Retired		8	2.51
*Referral criteria* (EDI-3-RF)				
	A least one		203	63.64
	None		116	36.36

**Table 2:** Pearson’s correlation, means, and standard deviations of the study variables.

	1	2	3	4	5	6
1. EAI	1					
2. EDI (F1)	0.292 **	1				
3. EDI (F2)	0.150 **	0.631 **	1			
4. EDI (F3)	0.172 **	0.638 **	0.449 **	1		
5. BICI	0.328 **	0.565 **	0.269 **	0.573 **	1	
6. RSES	**−** 0.203 **	−0.396 **	−0.230 **	−0.465 **	−0.631 **	1

**. Correlation is significant at the *p* < 0.01 level (two-tailed). EAI: Exercise Addiction Inventory; EDI (F1) = Drive for Thinness (Eating Disorder Inventory 3—Referral Form); EDI (F2) = Bulimia (Eating Disorder Inventory 3—Referral Form); EDI (F3) = Body Dissatisfaction (Eating Disorder Inventory 3—Referral Form); BICI = Body Image Concern Inventory; RSES = Rosenberg Self-Esteem Scale. Figures in bold represent significant relationships.

**Table 3:** Summaries of the models.

Antecedent	Total Effect [95% CI]	Direct Effect [95% CI]	Test of Highest Order Unconditional Interaction:	Bootstrapping 95% CI for the Moderation Effect
Drive for thinness	0.130 *** [0.0826; 0.1765]	0.069 * [0.0136; 0.1252]	Δ*R^2^* = 0.010 *F*(1, 315) = 6.810, *p* < 0.01	[−0.0418; −0.0043]
Bulimia	0.089 ** [0.0239; 0.1530]	0.039 [−0.0248; 0.1032]	Δ*R^2^* = 0.019 *F*(1, 315) = 10.252, *p* < 0.01	[−0.0676; −0.00129]
Body dissatisfaction	0.095 ** [0.0350; 0.1533]	−0.013 [−0.0881; 0.0699]	Δ*R^2^* = 0.016 *F*(1, 315) = 10.315, *p* < 0.01	[−0.0641; −0.0138]

*** *p* < 0.001; ** *p* < 0.01; * *p* < 0.05. Drive For Thinness: the relationship between Drive for Thinness and Exercise Addiction, with Body Image Concerns as mediator and Self-Esteem as moderator; Bulimia: the relationship between Bulimia and Exercise Addiction, with Body Image Concerns as mediator and Self-Esteem as moderator; Body Dissatisfaction: the relationship between Body Dissatisfaction and Exercise Addiction, with Body Image Concerns as mediator and Self-Esteem as moderator.

## Data Availability

The data presented in this study are available on request from the corresponding author. The data are not publicly available due to privacy reasons.
